# Mortality of Philadelphia, for April, May and June, 1852, Arranged from the Record Kept at the Health Office

**Published:** 1852-08

**Authors:** Wilson Jewell


*Mortality of Philadelphia, for April, May and June,* 1852, *arranged from the Record kept at the Health Office. With remarks on the new "Registration Law."* By WILSON JEWELL, M. D.

The second quarter of the present year, commenced on the 28th day of March, and terminated with the 3d of July, ult., embracing a period of 14 weeks and 1 day, or 99 days. According to the returns found at the Health Office, the number of deaths during the quarter, from all causes, amounted to 2634, showing a falling off of 151, or 6 per cent., from those of the first quarter of the year.

TABLE NO. 1 Exhibits a classification of the various causes of death during the quarter, together with an enumeration of the sexes, as well as the number of deaths for each month separately.

Comparing this table, with the same one for the first quarter of the year, as published in the June No. of this work, it will be found that the principal falling off has taken place among the deaths from diseases of the organs of respiration, amounting to 164, or 22 per cent.

TABLE NO. 2 Furnishes an accurate account of the names of the diseases found on the Register at the Health Office, being a faithful transcript from the certificates of the physicians. This nomenclature, which at the best is an imperfect one, we have not disturbed, preferring to allow each physician to speak for himself, rather than alter his phraseology, in order to show, how difficult it is to compile a statistical abstract, which would approach towards accuracy, in making comparisons of the prevalence of different diseases at distinct periods of the year, while this want of uniformity in attaching names to diseases shall continue to be observed.

This table also includes the number of deaths from the various causes assigned therefore, at fifteen distinct periods of life.

The deaths from Consumption of the Lungs as usual, occupy a large space in the table, amounting to 324; 175 of which took place between the ages of 20 and 24. The deaths from Consumption for the past six months have exceeded those for the same period of 1851, by 221—to wit:

| | 1851. | 1852. | | |
|---|---|---|---|---|
| 1st and 2d quarter, | 461 | 682 | Excess of 1852, | 221. |

Of the deaths recorded under the head of diseases of the Nervous System, amounting to 469, 315 took place in children under five years of age. The deaths from Convulsions alone, amounted to 131; 68, or more than half, occurring in children under one year of age.

The deaths from Scarlet Fever amounting to 98, have fallen off since the last quarter, about 35 per cent.

The mortality from Typhus and Typhoid Fevers has more than doubled itself sincc our last report. During the first quarter, January, February, and March, the deaths from these fevers numbered 48. The quarter just ended, shows 91 deaths, being an increase of 43.

Small Pox and Varioloid still continue to prevail. The deaths for this quarter number 137, two-thirds of which were under ten years of age, a striking evidence of the want of attention to vaccination among certain classes of our population. The deaths from Small Pox for the last six months, have amounted to 402; equal to two deaths per day.

By a reference to the tables in the June No. of the Examiner, it will be found that the deaths from Dysentery have doubled themselves. During the first quarter of the year, there were 34; while the second gives 75.

The deaths from Cholera Infantum have also increased during the present quarter. 44 cases of deaths are recorded, 40 of which were under two years of age.

Table No. 3 Presents the aggregate number of deaths during the second quarter, from all causes, for fifteen distinct periods of life. By this analysis, it will be seen that nearly half the deaths that have been registered, occurred in children under five years of age. Exclusive of the "Still Born," 551, or nearly one fourth were under one year of age.

Beyond the fifth year of life, the highest number of deaths took place between the ages of 30 and 40.

The deaths from "Still Born," 127; "External Causes," 72; "Old Age," 61; "Debility," 71, and "Unknown," 81, in all 412, will leave 2222 deaths from known diseases, which is equal to 22½ deaths per day, during the quarter.

TABLE NO. 1

*Deaths for the second quarter of* 1852, *classified.*

| | April | May. | June | Male | Fem. | Total. |
|---|---|---|---|---|---|---|
| 1. *Endemic and Contagious Diseases.* Zymotic or Epidemic, | 235 | 175 | 213 | 309 | 314 | 623 |
| 2. *Uncertain or general seat.* Sporadic Diseases | 108 | 87 | 120 | 173 | 142 | 315 |
| 3. Of the Nervous System, | 180 | 132 | 157 | 269 | 200 | 469 |
| 4. Organs of Respiration, | 248 | 190 | 146 | 314 | 270 | 584 |
| 5. " Circulation, | 28 | 16 | 26 | 34 | 36 | 70 |
| 6. Of the Digestive Organs, | 59 | 58 | 57 | 77 | 97 | 174 |
| 7. " Urinary " | 4 | 3 | 7 | 11 | 3 | 14 |
| 8. " Organs of Generation, | 14 | 7 | 3 | | 24 | 24 |
| 9. " Organs of Locomotion, | 3 | 6 | 5 | 7 | 7 | 14 |
| 10. " Integumentary System, | | 5 | 1 | 4 | 2 | 6 |
| 11. Old Age, | 25 | 18 | 18 | 20 | 41 | 61 |
| 12. Of External causes, | 19 | 21 | 32 | 55 | 17 | 72 |
| Still Born, | 53 | 26 | 48 | 70 | 57 | 127 |
| Unknown, | 23 | 25 | 33 | 39 | 42 | 81 |
| | 999 | 769 | 866 | 1382 | 1252 | 2634 |

TABLE No 2.

1. *Endemic and Contagious Diseases.—Zymotic or Epidemic.*

| | Male. | Female. | Under 1 yr. | 1 to 2. | 2 to 5. | 5 to 10. | 10 to 15. | 15 to 20. | 20 to 30. | 30 to 40. | 40 to 50. | 50 to 60. | 60 to 70. | 70 to 80. | 80 to 90. | 90 to 100. | 100 to 110. | Total. |
|---|---|---|---|---|---|---|---|---|---|---|---|---|---|---|---|---|---|---|
| Aphthæ . . . | 1 | 0 | 1 | 0 | 0 | 0 | 0 | 0 | 0 | 0 | 0 | 0 | 0 | 0 | 0 | | | 1 |
| Cholera Infantum . | 24 | 20 | 30 | 10 | 3 | 1 | 0 | 0 | 0 | 0 | 0 | 0 | 0 | 0 | 0 | | | 44 |
| " Morbus . . | 5 | 1 | 1 | 1 | 1 | 0 | 0 | 0 | 0 | 2 | 1 | 0 | 0 | 0 | 0 | | | 6 |
| Croup . . . | 23 | 25 | 5 | 14 | 24 | 5 | 0 | 0 | 0 | 0 | 0 | 0 | 0 | 0 | 0 | | | 48 |
| Diarrhœa . . . | 14 | 11 | 13 | 2 | 0 | 1 | 0 | 1 | 1 | 0 | 2 | 3 | 1 | 0 | 1 | | | 25 |
| Dysentery . . . | 39 | 36 | 10 | 15 | 16 | 3 | 1 | 0 | 6 | 4 | 6 | 5 | 5 | 3 | 1 | | | 75 |
| Erysipelas . . . | 8 | 16 | 7 | 1 | 2 | 0 | 0 | 0 | 0 | 3 | 4 | 3 | 2 | 2 | 0 | | | 24 |
| Fever . . . | 5 | 4 | 2 | 0 | 1 | 0 | 1 | 0 | 0 | 2 | 1 | 1 | 1 | 0 | 0 | | | 9 |
| " Congestive . . | 3 | 0 | 0 | 0 | 2 | 1 | 0 | 0 | 0 | 0 | 0 | 0 | 0 | 0 | 0 | | | 3 |
| " Hectic . . | 0 | 2 | 0 | 0 | 0 | 1 | 0 | 0 | 0 | 1 | 0 | 0 | 0 | 0 | 0 | | | 2 |
| " Inflammatory . | 0 | 1 | 0 | 0 | 0 | 0 | 0 | 0 | 0 | 1 | 0 | 0 | 0 | 0 | 0 | | | 1 |
| " Irritative . . | 1 | 0 | 1 | 0 | 0 | 0 | 0 | 0 | 0 | 0 | 0 | 0 | 0 | 0 | 0 | | | 1 |
| " Intermittent . | 1 | 0 | 0 | 0 | 0 | 0 | 0 | 0 | 1 | 0 | 0 | 0 | 0 | 0 | 0 | | | 1 |
| " Remittent . . | 5 | 5 | 0 | 1 | 2 | 2 | 0 | 0 | 0 | 0 | 0 | 1 | 4 | 0 | 0 | | | 10 |
| " Scarlet . . | 40 | 58 | 7 | 17 | 41 | 31 | 0 | 0 | 1 | 1 | 0 | 0 | 0 | 0 | 0 | | | 98 |
| " Synocha . . | 1 | 0 | 0 | 0 | 0 | 1 | 0 | 0 | 0 | 0 | 0 | 0 | 0 | 0 | 0 | | | 1 |
| " Typhus . . | 36 | 21 | 1 | 1 | 0 | 1 | 2 | 2 | 15 | 10 | 13 | 8 | 4 | 0 | 0 | | | 57 |
| " Typhoid . . | 18 | 16 | 0 | 0 | 0 | 3 | 0 | 2 | 9 | 11 | 3 | 1 | 5 | 0 | 0 | | | 34 |
| Hooping Cough . . | 0 | 2 | 1 | 0 | 1 | 0 | 0 | 0 | 0 | 0 | 0 | 0 | 0 | 0 | 0 | | | 2 |
| Measles . . . | 16 | 27 | 11 | 19 | 12 | 1 | 0 | 0 | 0 | 0 | 0 | 0 | 0 | 0 | 0 | | | 43 |
| Small Pox . . . | 67 | 67 | 22 | 17 | 36 | 15 | 4 | 4 | 23 | 10 | 1 | 1 | 0 | 1 | 0 | | | 134 |
| Syphilis . . . | 0 | 1 | 0 | 0 | 0 | 0 | 0 | 1 | 0 | 0 | 0 | 0 | 0 | 0 | 0 | | | 1 |
| Varioloid . . . | 2 | 1 | 2 | 0 | 0 | 1 | 0 | 0 | 0 | 0 | 0 | 0 | 0 | 0 | 0 | | | 3 |
| | 309 | 314 | 114 | 98 | 141 | 67 | 8 | 10 | 55 | 46 | 31 | 23 | 22 | 6 | 2 | 0 | 0 | 623 |

*2. Uncertain or General Seat.—Sporadic Diseases.*

| | Male. | Female. | Under 1 yr. | 1 to 2. | 2 to 5. | 5 to 10. | 10 to 15. | 15 to 20. | 20 to 30. | 30 to 40. | 40 to 50. | 50 to 60. | 60 to 70. | 70 to 80. | 80 to 90. | 90 to 100. | 100 to 110. | Total. |
|---|---|---|---|---|---|---|---|---|---|---|---|---|---|---|---|---|---|---|
| Abscess . . . | 2 | 2 | 0 | 1 | 0 | 0 | 0 | 0 | 1 | 0 | 2 | 0 | 0 | 0 | 0 | | | 4 |
| " of Neck . . | 1 | 0 | 0 | 0 | 0 | 0 | 0 | 0 | 0 | 0 | 1 | 0 | 0 | 0 | 0 | | | 1 |
| " of Thigh . . | 1 | 0 | 0 | 0 | 0 | 0 | 0 | 1 | 0 | 0 | 0 | 0 | 0 | 0 | 0 | | | 1 |
| Amputation of Leg . | 1 | 0 | 0 | 0 | 0 | 0 | 0 | 0 | 0 | 0 | 1 | 0 | 0 | 0 | 0 | | | 1 |
| Cancer . . . | 2 | 3 | 0 | 0 | 0 | 0 | 0 | 0 | 0 | 1 | 1 | 1 | 2 | 0 | 0 | | | 5 |
| Cachexia . . . | 6 | 0 | 3 | 0 | 0 | 1 | 0 | 0 | 1 | 0 | 0 | 1 | 0 | 0 | 0 | | | 6 |
| Congestion . . . | 0 | 1 | 0 | 1 | 0 | 0 | 0 | 0 | 0 | 0 | 0 | 0 | 0 | 0 | 0 | | | 1 |
| Cyanosis . . . | 3 | 7 | 8 | 0 | 1 | 0 | 0 | 1 | 0 | 0 | 0 | 0 | 0 | 0 | 0 | | | 10 |
| Cancer of Breast . . | 0 | 3 | 0 | 0 | 0 | 0 | 0 | 0 | 0 | 2 | 0 | 1 | 0 | 0 | 0 | | | 3 |
| Debility . . . | 36 | 35 | 33 | 2 | 2 | 1 | 0 | 2 | 1 | 6 | 9 | 8 | 5 | 1 | 1 | | | 71 |
| Disease of Throat . | 1 | 0 | 0 | 0 | 0 | 0 | 0 | 0 | 0 | 0 | 0 | 1 | 0 | 0 | 0 | | | 1 |
| Dropsy . . . | 20 | 16 | 0 | 1 | 5 | 4 | 2 | 2 | 0 | 4 | 2 | 7 | 3 | 5 | 1 | | | 36 |
| Effusion . . . | 1 | 0 | 0 | 0 | 1 | 0 | 0 | 0 | 0 | 0 | 0 | 0 | 0 | 0 | 0 | | | 1 |
| Gangrene . . . | 2 | 1 | 0 | 0 | 0 | 1 | 0 | 0 | 0 | 0 | 0 | 2 | 0 | 0 | 0 | | | 3 |
| " of Mouth . | 0 | 1 | 0 | 0 | 1 | 0 | 0 | 0 | 0 | 0 | 0 | 0 | 0 | 0 | 0 | | | 1 |
| Hemorrhage . . | 3 | 0 | 0 | 0 | 2 | 0 | 0 | 0 | 0 | 0 | 1 | 0 | 0 | 0 | 0 | | | 3 |
| " from Umbilicus | 1 | 1 | 1 | 1 | 0 | 0 | 0 | 0 | 0 | 0 | 0 | 0 | 0 | 0 | 0 | | | 2 |
| Inanition . . . | 14 | 11 | 23 | 0 | 1 | 0 | 0 | 0 | 0 | 0 | 0 | 0 | 0 | 0 | 1 | | | 25 |
| Inflammation . . | 0 | 2 | 1 | 0 | 0 | 0 | 0 | 0 | 1 | 0 | 0 | 0 | 0 | 0 | 0 | | | 2 |
| " Glandular | 1 | 1 | 1 | 1 | 0 | 0 | 0 | 0 | 0 | 0 | 0 | 0 | 0 | 0 | 0 | | | 2 |
| Injury of Perineum . | 1 | 0 | 0 | 0 | 0 | 0 | 0 | 0 | 0 | 0 | 0 | 1 | 0 | 0 | 0 | | | 1 |
| Malformation . . | 5 | 1 | 6 | 0 | 0 | 0 | 0 | 0 | 0 | 0 | 0 | 0 | 0 | 0 | 0 | | | 6 |
| " of Rectum | 1 | 0 | 1 | 0 | 0 | 0 | 0 | 0 | 0 | 0 | 0 | 0 | 0 | 0 | 0 | | | 1 |
| Marasmus . . . | 45 | 41 | 48 | 17 | 9 | 0 | 1 | 2 | 2 | 0 | 0 | 2 | 2 | 2 | 1 | | | 86 |
| Melœna . . . | 1 | 1 | 0 | 0 | 0 | 0 | 0 | 0 | 0 | 1 | 0 | 0 | 1 | 0 | 0 | | | 2 |
| Mortification . . | 2 | 0 | 0 | 0 | 0 | 0 | 0 | 0 | 0 | 0 | 0 | 0 | 0 | 1 | 1 | | | 2 |
| Scirrhus of Breast . | 0 | 1 | 0 | 0 | 0 | 0 | 0 | 0 | 0 | 0 | 0 | 1 | 0 | 0 | 0 | | | 1 |
| Scrofula . . . | 10 | 12 | 5 | 3 | 6 | 1 | 1 | 2 | 0 | 2 | 2 | 0 | 0 | 0 | 0 | | | 22 |
| Tabes Mesenterica . | 11 | 2 | 5 | 5 | 2 | 0 | 0 | 0 | 0 | 1 | 0 | 0 | 0 | 0 | 0 | | | 13 |
| Tumour of Neck . . | 1 | 0 | 0 | 0 | 0 | 0 | 0 | 0 | 0 | 0 | 1 | 0 | 0 | 0 | 0 | | | 1 |
| Ulceration . . . | 1 | 0 | 1 | 0 | 0 | 0 | 0 | 0 | 0 | 0 | 0 | 0 | 0 | 0 | 0 | | | 1 |
| | 173 | 142 | 136 | 32 | 30 | 8 | 4 | 10 | 6 | 17 | 20 | 25 | 13 | 9 | 5 | 0 | 0 | 315 |

### 3. *Of the Nervous System.*

| | Male. | Female. | Under 1 yr. | 1 to 2. | 2 to 5. | 5 to 10. | 10 to 15 | 15 to 20. | 20 to 30. | 30 to 40. | 40 to 50. | 50 to 60. | 60 to 70. | 70 to 80. | 80 to 90. | 90 to 100. | 100 to 110. | Total. |
|---|---|---|---|---|---|---|---|---|---|---|---|---|---|---|---|---|---|---|
| Ædema of Brain . . | 1 | 0 | 0 | 0 | 0 | 0 | 0 | 1 | 0 | 0 | 0 | 0 | 0 | 0 | 0 | | | 1 |
| Atrophy " . . | 0 | 1 | 1 | 0 | 0 | 0 | 0 | 0 | 0 | 0 | 0 | 0 | 0 | 0 | 0 | | | 1 |
| Apoplexy . . . | 12 | 11 | 0 | 0 | 0 | 0 | 0 | 1 | 1 | 6 | 5 | 2 | 6 | 0 | 1 | 1 | | 23 |
| Asthma Thymic . . | 1 | 0 | 0 | 1 | 0 | 0 | 0 | 0 | 0 | 0 | 0 | 0 | 0 | 0 | 0 | | | 1 |
| Cerebral Fever . . | 1 | 0 | 0 | 0 | 0 | 0 | 0 | 0 | 0 | 1 | 0 | 0 | 0 | 0 | 0 | | | 1 |
| Concussion of Brain . | 0 | 1 | 0 | 0 | 0 | 0 | 0 | 0 | 0 | 0 | 1 | 0 | 0 | 0 | 0 | | | 1 |
| Congestion " . . | 25 | 20 | 8 | 7 | 4 | 3 | 0 | 1 | 7 | 5 | 6 | 0 | 1 | 2 | 1 | | | 45 |
| Compression " . . | 0 | 1 | 0 | 0 | 0 | 0 | 0 | 0 | 1 | 0 | 0 | 0 | 0 | 0 | 0 | | | 1 |
| Coma . . . . | 0 | 1 | 0 | 0 | 0 | 0 | 0 | 0 | 0 | 0 | 0 | 0 | 0 | 1 | 0 | | | 1 |
| Convulsions . . | 71 | 60 | 68 | 29 | 21 | 5 | 2 | 1 | 1 | 1 | 2 | 1 | 0 | 0 | 0 | | | 131 |
| " Puerperal . | 0 | 5 | 0 | 0 | 0 | 0 | 0 | 2 | 2 | 1 | 0 | 0 | 0 | 0 | 0 | | | 5 |
| Cramp . . . . | 2 | 1 | 1 | 0 | 1 | 0 | 0 | 0 | 0 | 1 | 0 | 0 | 0 | 0 | 0 | | | 3 |
| Coup de Soliel . . | 3 | 0 | 0 | 0 | 0 | 0 | 0 | 0 | 2 | 1 | 0 | 0 | 0 | 0 | 0 | | | 3 |
| Disease of Brain . . | 15 | 9 | 11 | 5 | 1 | 3 | 1 | 0 | 0 | 1 | 0 | 1 | 1 | 0 | 0 | | | 24 |
| Dropsy " . . | 42 | 40 | 32 | 24 | 21 | 4 | 0 | 0 | 1 | 0 | 0 | 0 | 0 | 0 | 0 | | | 82 |
| Epilepsy . . . . | 8 | 0 | 0 | 0 | 0 | 0 | 1 | 1 | 1 | 2 | 3 | 0 | 0 | 0 | 0 | | | 8 |
| Effusion of Brain . | 16 | 12 | 3 | 13 | 4 | 0 | 1 | 0 | 0 | 1 | 2 | 2 | 2 | 0 | 0 | | | 28 |
| Inflammation of Brain . | 47 | 25 | 23 | 15 | 14 | 8 | 1 | 3 | 4 | 1 | 0 | 2 | 0 | 1 | 0 | | | 72 |
| Injury " . | 0 | 1 | 0 | 0 | 0 | 0 | 0 | 0 | 0 | 1 | 0 | 0 | 0 | 0 | 0 | | | 1 |
| Insanity . . . . | 1 | 0 | 0 | 0 | 0 | 0 | 0 | 0 | 0 | 0 | 0 | 1 | 0 | 0 | 0 | | | 1 |
| Mania . . . . | 1 | 0 | 0 | 0 | 0 | 0 | 0 | 0 | 0 | 0 | 1 | 0 | 0 | 0 | 0 | | | 1 |
| Meningitis . . . . | 1 | 0 | 1 | 0 | 0 | 0 | 0 | 0 | 0 | 0 | 0 | 0 | 0 | 0 | 0 | | | 1 |
| Mania a Potu . . . | 13 | 2 | 0 | 0 | 0 | 0 | 0 | 0 | 1 | 5 | 3 | 4 | 1 | 1 | 0 | | | 15 |
| Neurosis . . . . | 0 | 1 | 0 | 0 | 0 | 0 | 0 | 0 | 0 | 0 | 0 | 1 | 0 | 0 | 0 | | | 1 |
| Palsy . . . . . | 4 | 8 | 0 | 0 | 0 | 0 | 0 | 0 | 0 | 1 | 3 | 1 | 3 | 3 | 1 | | | 12 |
| Spasm of Glottis . . | 1 | 0 | 1 | 0 | 0 | 0 | 0 | 0 | 0 | 0 | 0 | 0 | 0 | 0 | 0 | | | 1 |
| Softening of Brain . | 0 | 1 | 0 | 0 | 0 | 0 | 0 | 0 | 0 | 1 | 0 | 0 | 0 | 0 | 0 | | | 1 |
| Tetanus . . . . | 1 | 0 | 0 | 0 | 0 | 0 | 1 | 0 | 0 | 0 | 0 | 0 | 0 | 0 | 0 | | | 1 |
| Trismus . . . . | 2 | 0 | 1 | 0 | 0 | 0 | 1 | 0 | 0 | 0 | 0 | 0 | 0 | 0 | 0 | | | 2 |
| Tuberculosis Cerebri . | 1 | 0 | 1 | 0 | 0 | 0 | 0 | 0 | 0 | 0 | 0 | 0 | 0 | 0 | 0 | | | 1 |
| | 269 | 200 | 151 | 94 | 66 | 23 | 8 | 10 | 21 | 29 | 26 | 15 | 14 | 8 | 3 | 1 | 0 | 469 |

### 4. *Organs of Respiration.*

| | Male. | Female. | Under 1 yr. | 1 to 2. | 2 to 5. | 5 to 10. | 10 to 15. | 15 to 20. | 20 to 30. | 30 to 40. | 40 to 50. | 50 to 60. | 60 to 70. | 70 to 80. | 80 to 90. | 90 to 100. | 100 to 110. | Total. |
|---|---|---|---|---|---|---|---|---|---|---|---|---|---|---|---|---|---|---|
| Abscess of Lungs . | | 2 | | | | | | | | | | 1 | | 1 | | | | 2 |
| Asthma . . . | 2 | 1 | | 1 | | | | | | | 1 | | | | 1 | | | 3 |
| Congestion of Lungs . | 8 | 5 | 7 | 2 | 1 | 1 | 1 | | | 1 | | | | | | | | 13 |
| Consumption " . | 165 | 159 | 7 | 4 | 7 | 4 | 11 | 25 | 84 | 91 | 45 | 32 | 9 | 3 | 1 | 1 | | 324 |
| Coryza . . . | 1 | | 1 | | | | | | | | | | | | | | | 1 |
| Disease of Lungs . | 3 | 3 | 1 | | | | 1 | | | | 1 | 1 | 1 | | 1 | | | 6 |
| " Chest . . | 1 | | | | | | | | | 1 | | | | | | | | 1 |
| Dropsy of Chest . . | 11 | 11 | 1 | 2 | 5 | 2 | 1 | 1 | 1 | 1 | 2 | 2 | 1 | 2 | 1 | | | 22 |
| Effusion " . . | 3 | | | | | | | | | | 1 | 2 | | | | | | 3 |
| " Lungs . . | 1 | | 1 | | | | | | | | | | | | | | | 1 |
| Gangrene " . . | 1 | | | | | | | 1 | | | | | | | | | | 1 |
| Hemorrhage " . . | 3 | 2 | | | | | | 1 | | | 3 | | 1 | | | | | 5 |
| Inflammation " . . | 70 | 52 | 29 | 22 | 18 | | 2 | | 11 | 8 | 10 | 4 | 14 | 2 | 2 | | | 122 |
| " Bronchia . | 34 | 24 | 20 | 14 | 7 | 4 | | | 3 | 1 | 2 | | 3 | 2 | 2 | | | 58 |
| " Chest . | | 1 | 1 | | | | | | | | | | | | | | | 1 |
| " Larynx . | 3 | 6 | 1 | 3 | 1 | | 1 | | | 1 | | 1 | 1 | | | | | 9 |
| " Pleura . | 2 | 1 | | | | | | 1 | 2 | | | | | | | | | 3 |
| " Throat . | 2 | 1 | 1 | 1 | 1 | | | | | | | | | | | | | 3 |
| " Tonsils . | 1 | | | | 1 | | | | | | | | | | | | | 1 |
| Palsy of Lungs . . | 1 | | | | | | | | | | 1 | | | | | | | 1 |
| Tuberculosis . . | 2 | 2 | 1 | 1 | | | 1 | | | 1 | | | | | | | | 4 |
| | 314 | 270 | 71 | 50 | 41 | 11 | 18 | 29 | 101 | 105 | 66 | 43 | 30 | 10 | 8 | 1 | 0 | 584 |

5. *Organs of Circulation.*

| | Male. | Female. | Under 1 yr. | 1 to 2. | 2 to 5. | 5 to 10. | 10 to 15. | 15 to 20. | 20 to 30. | 30 to 40. | 40 to 50. | 50 to 60. | 60 to 70. | 70 to 80. | 80 to 90. | 90 to 100. | 100 to 110. | Total. |
|---|---|---|---|---|---|---|---|---|---|---|---|---|---|---|---|---|---|---|
| Anæmia . . . . | 2 | 2 | 2 | | | | | | | 1 | | 1 | | | | | | 4 |
| Angina Pectoris . . | | 1 | | | | | | | | | | | 1 | | | | | 1 |
| Disease of Heart . . | 22 | 19 | 2 | | 1 | 4 | 3 | 1 | 4 | 5 | 7 | 3 | 6 | 4 | 1 | | | 41 |
| Dropsy " . . | 2 | 2 | | | | 1 | | | 1 | | | | 2 | | | | | 4 |
| Enlargement " . . | 3 | 2 | 1 | | | | | 1 | | 1 | 1 | 1 | | | | | | 5 |
| Inflammation of Heart . | 3 | 5 | 1 | | 2 | | | | 4 | 1 | | | | | | | | 8 |
| " of Vein . | 1 | | | | | | | 1 | | | | | | | | | | 1 |
| Malformation of Heart . | | 1 | 1 | | | | | | | | | | | | | | | 1 |
| Ossification " . | | 1 | | | | | | | | | | | | 1 | | | | 1 |
| " of Arteries | 1 | | | | | | | | | | | | | | 1 | | | 1 |
| Rheumatism of Heart . | | 3 | | | | | 1 | | | 2 | | | | | | | | 3 |
| | 34 | 36 | 7 | 0 | 3 | 5 | 4 | 3 | 9 | 10 | 8 | 5 | 9 | 5 | 2 | 0 | 0 | 70 |

6. *Digestive Organs.*

| | Male. | Female. | Under 1 yr. | 1 to 2. | 2 to 5. | 5 to 10. | 10 to 15. | 15 to 20. | 20 to 30. | 30 to 40. | 40 to 50. | 50 to 60. | 60 to 70. | 70 to 80. | 80 to 90. | 90 to 100. | 100 to 110. | Total. |
|---|---|---|---|---|---|---|---|---|---|---|---|---|---|---|---|---|---|---|
| Abscess of Liver . . | 0 | 1 | 0 | 0 | 0 | 0 | 0 | 0 | 1 | 0 | 0 | 0 | 0 | 0 | 0 | 0 | | 1 |
| Abdominal Dropsy . | 5 | 6 | 0 | 0 | 0 | 1 | 0 | 0 | 0 | 0 | 3 | 3 | 2 | 1 | 1 | 0 | | 11 |
| Cancer, Bowels . . | 0 | 1 | 0 | 0 | 0 | 0 | 0 | 0 | 0 | 0 | 1 | 0 | 0 | 0 | 0 | 0 | | 1 |
| " Liver . . | 0 | 1 | 0 | 0 | 0 | 0 | 0 | 0 | 0 | 0 | 0 | 1 | 0 | 0 | 0 | 0 | | 1 |
| " Rectum . . | 1 | 0 | 0 | 0 | 0 | 0 | 0 | 0 | 0 | 0 | 1 | 0 | 0 | 0 | 0 | 0 | | 1 |
| " Stomach . . | 0 | 3 | 0 | 0 | 0 | 0 | 0 | 0 | 0 | 0 | 1 | 0 | 2 | 0 | 0 | 0 | | 3 |
| Cancrum Oris . . | 2 | 4 | 1 | 1 | 2 | 2 | 0 | 0 | 0 | 0 | 0 | 0 | 0 | 0 | 0 | 0 | | 6 |
| Cirrhosus of Liver . | 0 | 1 | 0 | 0 | 0 | 0 | 0 | 0 | 0 | 0 | 0 | 0 | 0 | 1 | 0 | 0 | | 1 |
| Congestion " . . | 1 | 0 | 1 | 0 | 0 | 0 | 0 | 0 | 0 | 0 | 0 | 0 | 0 | 0 | 0 | 0 | | 1 |
| Colic . . . . | 2 | 2 | 2 | 0 | 0 | 0 | 0 | 0 | 0 | 0 | 1 | 0 | 0 | 1 | 0 | 0 | | 4 |
| Consumption of Bowels | 0 | 1 | 0 | 0 | 0 | 0 | 0 | 0 | 0 | 1 | 0 | 0 | 0 | 0 | 0 | 0 | | 1 |
| Disease " . . | 0 | 2 | 0 | 0 | 0 | 0 | 0 | 0 | 0 | 0 | 1 | 0 | 1 | 0 | 0 | 0 | | 2 |
| " Liver . . | 3 | 1 | 0 | 0 | 0 | 0 | 0 | 0 | 1 | 0 | 0 | 1 | 0 | 2 | 0 | 0 | | 4 |
| " Stomach . | 3 | 0 | 0 | 0 | 0 | 0 | 0 | 0 | 0 | 1 | 0 | 0 | 1 | 1 | 0 | 0 | | 3 |
| Dropsy of Liver . . | 0 | 1 | 0 | 0 | 0 | 0 | 0 | 0 | 0 | 0 | 0 | 0 | 0 | 0 | 1 | 0 | | 1 |
| Enlargem't " . . | 1 | 0 | 0 | 0 | 0 | 0 | 0 | 0 | 0 | 0 | 0 | 0 | 1 | 0 | 0 | 0 | | 1 |
| " Spleen . | 0 | 1 | 0 | 0 | 0 | 0 | 0 | 0 | 0 | 0 | 1 | 0 | 0 | 0 | 0 | 0 | | 1 |
| Gastric Fever . . | 0 | 1 | 0 | 0 | 0 | 0 | 0 | 0 | 1 | 0 | 0 | 0 | 0 | 0 | 0 | 0 | | 1 |
| Hemorrhage of Bowels | 1 | 1 | 0 | 0 | 0 | 0 | 0 | 1 | 1 | 0 | 0 | 0 | 0 | 0 | 0 | 0 | | 2 |
| Hernia . . . | 2 | 1 | 0 | 0 | 0 | 0 | 0 | 0 | 0 | 0 | 0 | 2 | 1 | 0 | 0 | 0 | | 3 |
| Ileus . . . . | 0 | 1 | 0 | 0 | 0 | 0 | 0 | 0 | 0 | 1 | 0 | 0 | 0 | 0 | 0 | 0 | | 1 |
| Indigestion . . . | 1 | 1 | 1 | 0 | 0 | 0 | 0 | 0 | 0 | 0 | 0 | 0 | 0 | 1 | 0 | 0 | | 2 |
| Inflam. Stomach & Bowels | 22 | 30 | 11 | 6 | 3 | 0 | 0 | 1 | 5 | 6 | 3 | 4 | 4 | 7 | 2 | 0 | | 52 |
| " Liver . . | 7 | 2 | 2 | 0 | 0 | 1 | 0 | 0 | 0 | 0 | 2 | 2 | 1 | 1 | 0 | 0 | | 9 |
| " Peritoneum . | 9 | 16 | 0 | 0 | 1 | 1 | 1 | 4 | 6 | 8 | 2 | 2 | 0 | 0 | 0 | 0 | | 25 |
| Intussusception . . | 2 | 1 | 2 | 0 | 1 | 0 | 0 | 0 | 0 | 0 | 0 | 0 | 0 | 0 | 0 | 0 | | 3 |
| Jaundice . . . | 2 | 3 | 3 | 0 | 0 | 0 | 0 | 0 | 0 | 1 | 0 | 1 | 0 | 0 | 0 | 0 | | 5 |
| Mortification Bowels . | 1 | 0 | 0 | 0 | 1 | 0 | 0 | 0 | 0 | 0 | 0 | 0 | 0 | 0 | 0 | 0 | | 1 |
| Obstruction Bowels . | 1 | 2 | 1 | 0 | 0 | 0 | 0 | 0 | 0 | 1 | 0 | 1 | 0 | 0 | 0 | 0 | | 3 |
| Perforation " . | 2 | 0 | 0 | 1 | 0 | 0 | 0 | 0 | 0 | 0 | 1 | 0 | 0 | 0 | 0 | 0 | | 2 |
| Scirrhus Pancreas . | 1 | 1 | 0 | 0 | 1 | 0 | 0 | 0 | 0 | 0 | 0 | 1 | 0 | 0 | 0 | 0 | | 2 |
| " Pylorus . . | 0 | 1 | 0 | 0 | 0 | 0 | 0 | 0 | 0 | 0 | 0 | 0 | 0 | 1 | 0 | 0 | | 1 |
| " Stomach . | 0 | 1 | 0 | 0 | 0 | 0 | 0 | 0 | 0 | 0 | 0 | 0 | 0 | 1 | 0 | 0 | | 1 |
| Softening Stomach . | 1 | 1 | 1 | 1 | 0 | 0 | 0 | 0 | 0 | 0 | 0 | 0 | 0 | 0 | 0 | 0 | | 2 |
| Teething . . . | 2 | 2 | 1 | 3 | 0 | 0 | 0 | 0 | 0 | 0 | 0 | 0 | 0 | 0 | 0 | 0 | | 4 |
| Ulceration of Bowels . | 3 | 3 | 0 | 0 | 0 | 0 | 0 | 1 | 1 | 1 | 1 | 1 | 0 | 1 | 0 | 0 | | 6 |
| " Stomach . | 1 | 1 | 0 | 0 | 0 | 0 | 0 | 0 | 0 | 1 | 0 | 0 | 0 | 1 | 0 | 0 | | 2 |
| " Throat . | 0 | 3 | 0 | 1 | 2 | 0 | 0 | 0 | 0 | 0 | 0 | 0 | 0 | 0 | 0 | 0 | | 3 |
| Worms . . . | 1 | 0 | 0 | 0 | 1 | 0 | 0 | 0 | 0 | 0 | 0 | 0 | 0 | 0 | 0 | 0 | | 1 |
| | 77 | 97 | 26 | 13 | 12 | 5 | 1 | 7 | 16 | 21 | 18 | 19 | 13 | 19 | 4 | 0 | 0 | 174 |

### 7. *The Urinary Organs.*

| | Male. | Female. | Under 1 yr. | 1 to 2. | 2 to 5. | 5 to 10. | 10 to 15. | 15 to 20. | 20 to 30. | 30 to 40. | 40 to 50. | 50 to 60. | 60 to 70. | 70 to 80. | 80 to 90. | 90 to 100. | 100 to 110. | Total. |
|---|---|---|---|---|---|---|---|---|---|---|---|---|---|---|---|---|---|---|
| Albuminuria . . | 1 | | | | | | | | | | | 1 | | | | | | 1 |
| Diabetes . . . | 2 | | | | | | | | | 1 | 1 | | | | | | | 2 |
| Disease of Kidneys . | 2 | 2 | | | 1 | 1 | | | 1 | | 1 | | | | | | | 4 |
| Inflam. of Bladder . | 1 | 1 | | | | 1 | | | | | 1 | | | | | | | 2 |
| " Kidneys . | 4 | | | | | | | | | | 2 | 1 | | | 1 | | | 4 |
| Stone . . . . . | 1 | | | | | | | | | | | | | 1 | | | | 1 |
| | 11 | 3 | 0 | 0 | 1 | 2 | 0 | 0 | 1 | 1 | 5 | 2 | 0 | 1 | 1 | 0 | 0 | 14 |

### 8. *Organs of Generation.*

| | Male. | Female. | Under 1 yr. | 1 to 2. | 2 to 5. | 5 to 10. | 10 to 15. | 15 to 20. | 20 to 30. | 30 to 40. | 40 to 50. | 50 to 60. | 60 to 70. | 70 to 80. | 80 to 90. | 90 to 100. | 100 to 110. | Total. |
|---|---|---|---|---|---|---|---|---|---|---|---|---|---|---|---|---|---|---|
| Cancer Uterus . . | | 3 | | | | | | | | | 1 | 1 | | | 1 | | | 3 |
| Chlorosis . . . | | 1 | | | | | | 1 | | | | | | | | | | 1 |
| Hemorrhage from Uterus | | 2 | | | | | | | | 2 | | | | | | | | 2 |
| Inflammation " | | 6 | | | | | | | | 6 | | | | | | | | 6 |
| Puerperal Fever . . | | 8 | | | | | | | 5 | 1 | 2 | | | | | | | 8 |
| " Mania . . | | 1 | | | | | | | 1 | | | | | | | | | 1 |
| Ovarian Dropsy . . | | 1 | | | | | | | 1 | | | | | | | | | 1 |
| " Tumor . . | | 2 | | | | | | | | | | | 1 | 1 | | | | 2 |
| | 0 | 24 | 0 | 0 | 0 | 0 | 0 | 1 | 7 | 9 | 3 | 1 | 1 | 1 | 1 | 0 | 0 | 24 |

### 9. *Organs of Locomotion.*

| | Male. | Female. | Under 1 yr. | 1 to 2. | 2 to 5. | 5 to 10. | 10 to 15. | 15 to 20. | 20 to 30. | 30 to 40. | 40 to 50. | 50 to 60. | 60 to 70. | 70 to 80. | 80 to 90. | 90 to 100. | 100 to 110. | Total. |
|---|---|---|---|---|---|---|---|---|---|---|---|---|---|---|---|---|---|---|
| Caries of Cranium . | 1 | | | | | | | | | 1 | | | | | | | | 1 |
| " Jaw . . | | 1 | | | | | | | | | | 1 | | | | | | 1 |
| " Spine . . | 1 | | | | | | | | | | | 1 | | | | | | 1 |
| Disease of Spine . . | 2 | 1 | | | 2 | 1 | | | | | | | | | | | | 3 |
| Effusion of Spinal Marrow | | 1 | | | | | | | | 1 | | | | | | | | 1 |
| Medul. Tumor Knee joint | | 1 | | | | | | | 1 | | | | | | | | | 1 |
| Rheumatism . . | 3 | 2 | | | | | 1 | | 1 | 1 | 1 | 1 | | | | | | 5 |
| Spinal Meningitis . | | 1 | | | 1 | | | | | | | | | | | | | 1 |
| | 7 | 7 | 0 | 0 | 3 | 1 | 1 | 0 | 2 | 3 | 1 | 3 | 0 | 0 | 0 | 0 | 0 | 14 |

10. *The Integumentary System.*

| | Male. | Female. | Under 1 yr. | 1 to 2. | 2 to 5. | 5 to 10. | 10 to 15. | 15 to 20. | 20 to 30. | 30 to 40. | 40 to 50. | 50 to 60. | 60 to 70. | 70 to 80. | 80 to 90. | 90 to 100. | 100 to 110. | Total. |
|---|---|---|---|---|---|---|---|---|---|---|---|---|---|---|---|---|---|---|
| Purpura . . . | 1 | 1 | 2 | | | | | | | | | | | | | | | 2 |
| Scorbutus . . . | 2 | | | | | | | | 2 | | | | | | | | | 2 |
| Suppressed Eruption . | 1 | | | | 1 | | | | | | | | | | | | | 1 |
| Ulcers . . . | | 1 | | | | | | | | | | | | 1 | | | | 1 |
| | 4 | 2 | 2 | 0 | 1 | 0 | 0 | 0 | 2 | 0 | 0 | 0 | 0 | 1 | 0 | 0 | 0 | 6 |

11. *Old Age.*

| | Male. | Female. | Under 1 yr. | 1 to 2. | 2 to 5. | 5 to 10. | 10 to 15. | 15 to 20. | 20 to 30. | 30 to 40. | 40 to 50. | 50 to 60. | 60 to 70. | 70 to 80. | 80 to 90. | 90 to 100. | 100 to 110. | Total. |
|---|---|---|---|---|---|---|---|---|---|---|---|---|---|---|---|---|---|---|
| Old Age . . . | 20 | 41 | | | | | | | | | | | 6 | 28 | 24 | 3 | | 61 |

12. *From External Causes.*

| | Male. | Female. | Under 1 yr. | 1 to 2. | 2 to 5. | 5 to 10. | 10 to 15. | 15 to 20. | 20 to 30. | 30 to 40. | 40 to 50. | 50 to 60. | 60 to 70. | 70 to 80. | 80 to 90. | 90 to 100. | 100 to 110. | Total. |
|---|---|---|---|---|---|---|---|---|---|---|---|---|---|---|---|---|---|---|
| Asphyxia . . . | 3 | 3 | 6 | | | | | | | | | | | | | | | 6 |
| Burns and Scalds . . | 3 | 2 | | 3 | 1 | 1 | | | | | | | | | | | | 5 |
| Casualties . . . | 13 | 2 | 1 | 1 | | 2 | 1 | 1 | 6 | 2 | | 1 | | | | | | 15 |
| Drowned . . . | 24 | 3 | | | 1 | 2 | 1 | 2 | 6 | 9 | 6 | | | | | | | 27 |
| Exposure . . . | 1 | | | | | | | | | | | | 1 | | | | | 1 |
| Fracture Leg . . | 1 | | | | | | | | 1 | | | | | | | | | 1 |
| " Pelvis . . | 1 | | | | | | | | 1 | | | | | | | | | 1 |
| " Skull . . | 1 | | | | | | | | | 1 | | | | | | | | 1 |
| Gun-shot Wound . . | 1 | | | | | | | | 1 | | | | | | | | | 1 |
| Intemperance . . | 2 | 4 | | | | | | | 1 | 4 | 1 | | | | | | | 6 |
| Suffocation . . . | 1 | 3 | 3 | | | | | | | | | 1 | | | | | | 4 |
| Suicide . . . | 3 | | | | | | | | 1 | | 2 | | | | | | | 3 |
| Wound . . . | 1 | | | | | | | | 1 | | | | | | | | | 1 |
| | 55 | 17 | 10 | 4 | 2 | 5 | 2 | 3 | 18 | 16 | 9 | 2 | 1 | 0 | 0 | 0 | 0 | 72 |
| Unknown . . . | 39 | 42 | 34 | 3 | 5 | | | | 8 | 6 | 11 | 7 | 3 | 2 | 2 | | | 81 |
| Still Born . . . | 70 | 57 | 127 | | | | | | | | | | | | | | | 127 |

TABLE NO. 3.

*Deaths for the second quarter, at fifteen distinct periods of life.*

| | |
|---|---|
| Under 1 year, | 551 |
| 1 to 2 | 294 |
| 2 to 5 | 305 |
| 5 to 10 | 127 |
| 10 to 15 | 46 |
| 15 to 20 | 73 |
| 20 to 30 | 246 |
| 30 to 40 | 263 |
| 40 to 50 | 198 |
| 50 to 60 | 145 |
| 60 to 70 | 112 |
| 70 to 80 | 90 |
| 80 to 90 | 52 |
| 90 to 100 | 5 |
| 100 to 110 | 0 |
| | 2507 |
| Still Born | 127 |
| Total, | 2634 |

Among the deaths during the quarter, there were from the almshouse 237; people of color, 214; and from the country, 31, as follows:

| | Blacks. | Almshouse. | Country. |
|---|---|---|---|
| April, | 70 | 72 | 11 |
| May, | 61 | 82 | 9 |
| June, | 83 | 83 | 11 |
| Total, | 214 | 237 | 31 |

TABLE NO. 4.

*Meteorological Summary.*

| | TEMPERATURE. | | | SNOW AND RAIN. |
|---|---|---|---|---|
| | Highest. | Lowest. | Mean. | Inches. |
| April | 52.6° | 40.6° | 46 6° | 6.445 |
| May | 71.5 | 55.1 | 63.3 | 3.034 |
| June | 79.7 | 63.8 | 71.7 | 4.030 |

### *The new Registration Law of Marriages, Births, and Deaths.*

With the expiration of the present or second quarter of the year, the new "Registration Law" goes into operation throughout the State. All the business, therefore, growing out of the collection and arrangement of the births and deaths in the city of Philadelphia, has been taken from the hands of the Board of Health, and their efficient and gentlemanly clerk, Samuel P. Marks, Esq., who, for many years, has catered to the entire satisfaction of the profession in these matters, and has been transferred to the office of the "Register of Wills," which officer, has been appointed to carry out the details of the new law, in Philadelphia City and County.

This it will be perceived, is an important change, embracing a variety of new and interesting features, and involving numerous perplexities at the outset. Should the accumulated machinery, that will be required, in order to conduct the enlarged system of registration as proposed in this law, not work with regularity at the commencement, more than usual allowance will have to be granted by those interested in the provisions of the act, until the new officers, who are mere novices in the business, become familiar with their arduous duties. Already, have difficulties and inconveniences arisen to hinder the progress of the measure, and we opine that it will occupy an unlimited period, before uniformity and accuracy in securing and registering Births, Marriages, and Deaths, can be successfully effected.

It is well ascertained that the "Registration law" is by no means a popular measure in the State, as strong efforts were made to defeat the passage of the act in the Legislature. In the interior and elsewhere there are those at this day who would be gratified if it should fail to be carried out. We are not surprised, therefore, to find lukewarmness and tardiness prevailing in those quarters, where we should look for efficient action.

Like all laws of a general character, or, to use a legislative phrase, like all "fancy bills" having no favorite political object to accomplish, nor in any manner affecting the private interests of certain wealthy corporations, there is nothing provided to warm it into existence, and it is obliged to rely upon the cold and disinterested charities of strangers, who, though they have no individual interests to gratify, volunteer to furnish it with healthful nourishment.

We know not otherwise, how to account for the strange and unnecessary delay at Harrisburg in providing the proper forms for the Registers' offices throughout the State. Up to this period, July 22d, the Office in this city, where above all others they are highly necessary, owing to the rapid and constant accumulation of business, the Register, has not received a single form Book from the Secretary of the Commonwealth; and if we are correctly informed, these books are not yet printed.

Nor has public notice to any extent been given of the precise time when this law goes into operation, nor has any extended information been published, as to the duties and obligations required of those, from whom the information is to be derived.

We speak advisedly for Philadelphia County when we assert, that many of our undertakers, sextons, clergymen, and even physicians and magistrates, have to plead entire ignorance of the requirements of this law, as well as for its existence; so limited has been public information on the subject.

Since the 3d of July, up to which date the Board of Health published their weekly statement of deaths, the Board have declined to receive certificates of Births and Deaths, while the Register of Wills, who has not been fully inducted into this special department of his office, not having had any preparation made for him at Harrisburg to register the returns, has necessarily allowed the whole business to remain a chaotic mass.

We have to regret, that in consequence of the change effected by the operation of this new registration law, we are in danger of losing the weekly statement of deaths, which for years we have been accustomed to receive from the Health Office; nor are we able, from our present information on this subject, to comprehend, or to learn with satisfaction, how or when we shall receive any public account of the births and deaths in our city, other than what is contained in the 13th section of the law; where we are told, that each Register furnishes the Secretary of the Commonwealth with a semi-annual statement, who files the same in his office, and annually transmits to the Legislature an abstract of the births, marriages, and deaths, which have occurred in each county in the State during the year. Consequently, if we are to be supplied with a statement, of the weekly, monthly or quarterly returns of deaths hereafter, we must rely upon private and irresponsible evidence and enterprise; unless the Register can be prevailed upon as a favor, to present a weekly table of the diseases and deaths therefrom, as has been done heretofore. If done at all it must be a favor, as no provision is contained in the law for any other public statement, than that to be made annually through the Legislature.

There are many features in this law, which we could have desired to have been otherwise expressed, but we wish to be satisfied with it as it is, until we have an opportunity to observe its operation, hoping that it may give general satisfaction and eventually confer a favor on the masses. We cannot, however, conceal the fact, that there are many hindrances to its progress; but we know of none, nor need we allude to any other in this connection which ought to cause greater solicitude than the indifference with which intelligent members of our profession treat this important measure. They speak of it as an onerous law, they regard the collecting of statistics, as is provided for in this act, both useless and unnecessary, while at the same time they view the whole plan as one impracticable in its character throughout the commonwealth.

It is true, the change from the old system may burden us with a little more labor, and occupy a little more of our time, than we have been accustomed to allot to the work of furnishing our certificates of births and deaths, but then the object is of such vital importance, that we cannot but hope every medical man who is inclined to find fault with the law, will waive his objections for the general good, as well as for the character of the profession of which he is a member, and give it his hearty approval and support.

We have always entertained the sentiment that the members of the medical profession above all others, should be and would be the firm advocates of a uniform and systematic registration of births, marriages, and deaths; that they are more competent to appreciate its advantages and to be profited by its invaluable results. If the mass of our profession does not sustain this law, who we inquire, will promote its claims? Its successful accomplishment rests with us. The intelligent public, the political economist, the vital statist, and the profession in those States where a system of registration has been introduced and proved successful, are looking on with deep interest to behold the result of a similar measure in this State. Shall they be disappointed?

On the other hand there are those in our own commonwealth, and we hope they are not many, who have from the beginning opposed the passage of this registration law, who will no doubt exult if it should prove a failure, and a failure too from the supineness or negligence of that profession who should of all others prove its warmest admirers, its undeviating supporters and its best friends.

We sincerely trust, therefore, that the profession throughout the State will, as far as their means and opportunities may enable them, favor in every possible way its claims and its progress, sacrificing private judgment, whenever it can be done without a violation of the conscience, for the general good.

